# Outcome of head injury in motorbike riders

**DOI:** 10.12669/pjms.39.2.6371

**Published:** 2023

**Authors:** Shakeel Ahmad, Lal Rehman, Ali Afzal, Farrukh Javeed

**Affiliations:** 1 Dr. Shakeel Ahmad, FCPS., Department of Neurosurgery, Jinnah Postgraduate Medical Center, Rafiqui Shaheed Road, Karachi, Pakistan; 2 Dr. Lal Rehman, FRCS., Department of Neurosurgery, Jinnah Postgraduate Medical Center, Rafiqui Shaheed Road, Karachi, Pakistan; 3 Dr. Ali Afzal, FCPS., Department of Neurosurgery, Nottingham University Hospital, Nottingham, UK; 4 Dr. Farrukh Javeed, FCPS., Department of Neurosurgery, Jinnah Postgraduate Medical Center, Rafiqui Shaheed Road, Karachi, Pakistan

**Keywords:** Motorbike accidents, Helmet use, TBI, GCS, GOS, RTA

## Abstract

**Objective:**

To determine the impact of helmet wearing on traumatic brain injury.

**Methods:**

We analyzed 400 cases of traumatic brain injury (TBI) in motorbike riders with and without helmet, from July 2017 to December 2020 presenting to the neurosurgery department at Jinnah Postgraduate Medical Center (JPMC), Karachi, Pakistan. The medical records were analyzed for CT scan findings, length of hospital stay, complications (mortality and disability), Glasgow Coma Scale (GCS) and Glasgow outcome score (GOS) at time of discharge.

**Result:**

A total of 400 patients with head injury due to motorbike accidents were included and all were male patients. They were equally divided into two groups, 200 in Group-A (with helmet) and 200 in Group-B (without helmet). Majority of the unhelmeted patients i.e. 102 (51%), needed admission in the Intensive Care Unit (ICU) compared to 70 (35%) in helmeted. When comparing non-helmeted patients to helmeted patients, the total median length of hospital stay was greater among non-helmeted patients (10 vs 05 days). Mortality was higher among non-helmeted patients seen in 50 (25%) as compared to 14 (7%) in helmeted patients. Overall, the good outcome was observed in 119 (59.5%) patients in Group-A as compared to70 (35%) patients in Group-B while 81 (40.5%) showed bad outcome in Group-A and 130 (64%) in Group-B. The failure to wear a helmet was found to be strongly linked with abnormal neuroimaging more complications, poor outcome and lower GCS on discharge as compared to patients using helmet.

**Conclusion:**

Lack of helmet use is linked to abnormal brain imaging, more complications, and a longer stay in the hospital after a head injury.

## INTRODUCTION

Motorbike accident is a leading global cause of unnatural death. Nearly half of all traffic fatalities occur among those with the least protection: highest observed with motorcyclists (23%), pedestrians (22%), and cyclists (4%).^1^ Motorbike riders are more susceptible to injury if involved in a collision and are reported to suffer a 26 times increased risk of death in a crash than the drivers riding other types of vehicles.^2^,^3^ Motorcycle accidents account for 8-19% of road accident deaths and the majority of deaths are secondary to head trauma, which is estimated to be responsible for more than 80% of the casualties in low- and middle-income countries.^4^,^5^

According to one study, the employment rate before sustaining a moderate or severe TBI was 80%, but the rate was just 15% three months after injury, and the rate had only increased to 55% three years following injury.^6^ The use of motorcycle helmets has been shown to be helpful in reducing the risk of death and head injury in motorcycle crashes.^7^,^8^ The aim of study was to find out the effectiveness of helmet use and to highlight its importance in reducing TBI in motorbike riders.

## METHODS

This is an observational study where 400 motorbike riders were included from July 2017 to December 2020, presenting with head injuries, to the department of neurosurgery at Jinnah Postgraduate Medical Center, Karachi, Pakistan. All participants or their families in cases of unconscious patients, provided informed consent and the study was conducted with Institutional Review Board (IRB) permission (Reference No# 226, 20^th^ July 2022). Patients involved in motorcycle accidents were initially assessed, resuscitated, and managed appropriately for their injuries at the emergency department. All patients with head injuries who needed to be admitted and treated had their demographic and clinical information gathered. Police and/or paramedic records helped us identify whether the motorbike rider was wearing a helmet at the time of the accident or not. This study did not include pedestrians and automobile occupants who were involved in motorbike collisions. Children, patients whose helmet use could not be determined, and patients who did not sustain a head injury were also excluded from the study.

The severity of the head injury was classified as mild, moderate, or severe based on the GCS score. CT scan was done and patients were observed for complications. The data about demographic information, helmet use, GCS at admission, CT scan findings, hospital stay and ICU admission, complications including mortality and GCS upon discharge and outcome at discharge were all collected. Outcome was calculated in terms of Glasgow outcome score (GOS) which was taken as Good outcome (GOS 5; good recovery) and Bad outcome (GOS 1; death, GOS 2; vegetative state, GOS 3; severe disability and GOS 4; moderate disability). Mean SD (standard deviation) and percentage (percentage) were used to express all of the data. The t test was used to check the statistical significance of differences. A p value of less than 0.05 was considered to be statistically significant.

## RESULTS

In this study, 400 patients with head injury due to motorbike accidents were included and all were male. These patients were split into two groups, 200 in Group-A (with helmet) and 200 in Group-B (without helmet). The youngest patient included was 15 years old while the oldest was 65 years old. Motorbike accident was common in younger age group (15-30 years) comprising 160 (40%) out of 400 patients and most of them 110 (55%) belonged to Group-B. In Group-A, extra axial hematomas (epidural, subdural and subarachnoid hemorrhages) were seen in 42 (22%) patients, intraparenchymal hemorrhage including intraventricular hemorrhage and contusions in 37 (18.5%), skull fracture in 14 (7%), diffuse axonal injury in 19 (9.5%) while in Group-B epidural, subdural and subarachnoid hemorrhages was seen in 55 (27.5%) patients, intraparenchymal hemorrhage in 48 (24%), skull fracture in 31 (15.5%), and diffuse axonal injury in 23 (115%) as shown in Table-I.

**Table-I:** Demographic data of patients.

S No.	Characteristics		Group-A (%)	Group-B (%)
	CT Scan Findings	Unremarkable	88 (44)	43 (21.5)
		Skull fracture	14 (7)	31 (15.5)
		Extra-axial hematoma	42 (22)	55 (27.5)
		Intra-parenchymal hematoma	37 (18.5)	48 (24)
		Diffuse axonal injury	19 (9.5)	23 (11.5)
	Complications	Fits	22 (11)	38 (19)
		Meningitis	06 (3)	10 (5)
		CSF leak	08 (4)	13 (6.5)
		Chest infection	14 (7)	21 (10.5)
		Total	40 (20)	82 (41)
	Mortality		14 (7)	50 (25)
	Hospital stay (number of days)		05	10
	ICU admission		70 (35)	102 (51)

In Group-A, complications developed in 40 (20%) patients, namely fits in 22 (11%), meningitis in 06 (3%), CSF leak in 08 (4%) and 14 (7%) developed chest infection while in Group-B complications were seen in 82 (41%) patients with fits in 38 (19%), meningitis in 10 (5%), CSF leak in 13 (6.5%) and chest infection in 21 (10.5%) patients, represented in Table-I. Majority of the unhelmeted patients were admitted in the ICU (51%) as compared to 35% in helmeted. When comparing non-helmeted patients to helmeted patients, the total median length of hospital stay was greater among non-helmeted patients (10 vs 05 days). Mortality was higher among non-helmeted patients, seen in 50 (25%) as compared to 14 (7%) in helmeted patients shown in Table-I.

The GCS at time of presentation, GCS at discharge and GOS at discharge were calculated for all patients. In Group-A, GCS at the time of presentation was mild (13-15) in 126 (63%) patients, moderate (9-12) in 48 (24%) and severe (3-8) in 22 (11%), which improved at the time of discharge to 13-15 in 133 (71.5%) patients, 9-12 in 26 (13.9%) and 3-8 in 27 (14.5%) patients given in Table-I. While, in Group-B, GCS at presentation was 13-15 in 98 (49%) patients, 9-12 in 66 (33%) and 3-8 in 36 (18%), and at the time of discharge GCS was 13-15 in 91 (60.7%) patients, 9-12 in 18 (12%) and 3-8 in 41 (27.3%). According to GOS, 119 (59.5%) patients showed good outcome in Group-A and 70 (35%) patients in Group-B while 81 (40.5%) showed bad outcome in Group-A and 130 (64%) in Group-B, as shown in Table-II.

**Table-II:** Comparison GCS on presentation and discharge between two groups.

Variables	Group-A (%)	Group-B (%)	P value*
***GCS on presentation***			0.001
13 – 15	126 (63)	98 (49)	
9 – 12	48 (24)	66 (33)	
3 – 8	22 (11)	36 (18)	
Total	200	200	
***GCS on discharge***			0.014
13 – 15	133 (71.5)	91 (60.7)	
9 – 12	26 (13.9)	18 (12)	
3 – 8	27 (14.5)	41 (27.3)	
Total	186	150
***GOS***			< 0.000
Good outcome	119 (59.5)	70 (35)	
Bad outcome	81 (40.5)	130 (65)	
Total	200	200	

* Significant = <0.05, Insignificant = >0.05.

## DISCUSSION

Many research demonstrating the preventative effectiveness of helmets against the effects of impact on human skull models have proven the relation between helmet wear and head injury severity. Our findings show that not wearing a helmet is strongly linked to higher abnormal head CT findings, complications, and poor outcomes. Helmet wearing has been demonstrated to minimize the incidence of brain injury by 88% in studies.^9^ Similar to prior studies, younger patients were more frequently seen without a helmet in our study, 110 (55%) patients in Group-B as compared to 50 patients in Group-A patients.^10^-^12^ The lack of helmet use among the young may be attributable to the fact that young individuals, in comparison to older persons, are more likely to engage in risky activities and attitudes.^13^

Wearing a helmet can lower the acceleration experienced by up to 87% during impact and can help the skull endure forces up to 47 pounds, according to a study utilizing human cadaver skulls.^14^ The current study’s finding that non-use of a helmet was strongly related with abnormal CT scans concurs with two studies conducted in the United States.^4^,^15^ There may be a connection between the high rate of abnormal CT scan findings among non-helmeted patients and the fact that motorcycle riders who do not wear helmets are at a much higher risk of sustaining head and traumatic brain injuries, which can manifest themselves as abnormal CT scan findings such as fractures, hematomas, contusions, and brain haemorrhage, among other things.

The incidence of TBI was also significantly higher among unhelmeted bike riders of any type of vehicle. For moped riders, the incidence of TBI among unhelmeted motorbike riders was 60% higher than helmeted motorbike riders.^3^ The use of helmets provides an additional layer of protection for the rider’s head, reducing the incidence of serious kinds of traumatic brain injury. In our study, in patients using helmet, CT scan abnormalities like extra axial hematomas were seen in 66 (33%) patients while, skull fracture in 20 (10%) and this ratio was significantly lower than patients not using helmet with 55 (27.5%) extra-axial hematomas and 31 (15.5%) skull fractures. According to Chalya et al, the incidence of skull fractures was nearly six times greater for unhelmeted motorbike riders while in our study, skull fractures were observed two times more common in Group-B (without helmets) than Group-A (with helmets) patients.^14^

Use of helmet also affects duration of hospitalization. The failure to wear a helmet is strongly connected with more severe TBI and the need to be taken to the hospital.^16^ Compared to those who wore helmets, patients who did not wear helmets spent more time in the hospital, according to one study.^9^ In our study, non-helmeted patients had a longer overall median hospitalization than helmeted patients. The longer hospital stay of non-helmeted patients could be explained by the fact that the majority of motorcycle riders who did not wear a helmet received serious traumatic brain injuries, necessitating protracted hospitalization.

Both moderate and severe TBI patients need to be kept in ICU for management and continuous neuro observation. Unhelmeted motorbike riders sustain more severe head injury which results in long ICU stays and increase in hospital charges which is consistent with other recently published studies.^9^,^17^,^18^ In our study, 172 patients from both groups were admitted to ICU, 102 (51%) unhelmeted compared to 70 (35%) helmeted, which shows a significant association with the need for ICU admission among patients without helmets.

Many complications can occur in TBI patients like infections, fits, neurological deficit and even death. Choi WS et al studied data from 23 university hospital in South Korea on motorcycle riders to show that the fatality rate was almost three times higher in unhelmeted compared to helmeted motorbike riders.^19^ In comparison to non-helmeted motorcyclists, it has been found that the usage of motorcycle helmets decreases the overall death rate in motorcycle accidents.^7^,^20^,^21^ Hofmann LJ et al conducted a survey asking surgeons who were members of the American Association for the Surgery of Trauma (AAST) about the helmet use and they quoted that motorbike related mortality can be reduced by 35% with use of helmet.^22^ In our study in Group-A patients showed 40 (20%) complication as compared to 82 (41%) in Group-B. Group-A patients had fits in 22 (11%) patients, meningitis in 06 (3%), CSF leak in 08 (4%) and chest infection in 14 (7%) patients while Group-B patients had fits in 38 (19%) patients, meningitis in 10 (5%), CSF leak in 13 (6.5%) and chest infection in 21 (10.5%) patients. The GOS was also a major difference in both the groups where 59.5% patients showed good outcome in helmeted patients while only 35% non-helmet patients recovered to good outcome which was also statistically significant (p value <0.05).

The overall mortality occurred in 64 (16%) patients, but it was significantly higher (25%) in patients without helmets than 7% in helmeted demonstrating the significance of motorcycle helmets in prevention of deaths among motorcycle accident patients. A study from Thailand shows that the outcomes were better with the implementation of law to use helmet and mortality among motorbike users would immediately come down by 23% if helmet use increases to 90% from 44% in 2010.^7^ We believe the high mortality rate among non-helmeted patients in our study can be accounted by the fact that the vast majority of non-helmeted patients suffered more severe trauma and brain injuries, both of which were proven to be predictive of death in the present study’s participants.

When comparing patients who wore helmets to those who did not, the outcome was significantly better in the former group. Direct remedies such as these could increase the mobility of motorcycle riders while also reducing their susceptibility in the pre-crash period. Having little or no motorcycle riding experience is thought to be connected with an increased risk of motorcycle accidents and injuries on a regular basis. This population is likely to benefit from formal driver training, which will improve their riding skills and lower their likelihood of motorcycle injuries. However, there has been some debate over the benefits of training courses, since riders who got instruction were reported to have had no substantial reduction in their likelihood of being involved in a motorcycle accident compared to those who did not.^3^

### Limitations

This study was confined to a small number of patients and a single site; a larger sample size and the participation of other centers would undoubtedly provide a more substantial insight into the topic.

## CONCLUSION

Lack of helmet use leads to increased hospital admissions with life threatening head injuries mandating intensive care, whereas the use of helmet significantly reduces the severity of head injury, hospitalization duration, morbidity and mortality. As a result, it is vital to organize public awareness campaigns on the safety benefits of wearing helmets, as well as consistent enforcement of traffic laws, in assuring compliance and a shift in views.

### Authors’ Contribution:

**SA** conceived and designed the study.

**AA** did data collection and manuscript writing.

**FJ** did statistical analysis & editing of manuscript, is accountable for integrity of the study.

**LR** did review and final approval of manuscript.
